# Characteristics associated with clinical response to Comano thermal spring water balneotherapy in pediatric patients with atopic dermatitis

**DOI:** 10.1186/s13052-021-00971-3

**Published:** 2021-04-16

**Authors:** Davide Geat, Mattia Giovannini, Ezio Gabriele Barlocco, Riccardo Pertile, Stefania Farina, Manuela Pace, Cesare Filippeschi, Giampiero Girolomoni, Mario Cristofolini, Ermanno Baldo

**Affiliations:** 1 Department of Medicine, Section of Dermatology and Venerology, University of Verona, Verona, Italy; 2 Allergy Unit, Department of Pediatrics, Meyer Children’s University Hospital, Florence, Italy; 3 “Giovan Battista Mattei” Research Institute, Stenico, Italy; 4 Department of Clinical and Evaluative Epidemiology, Trento Health Service, Trento, Italy; 5 Department of Pediatrics, S. Maria del Carmine Hospital, Rovereto, Italy; 6 Dermatology Unit, Department of Pediatrics, Meyer Children’s University Hospital, Florence, Italy

**Keywords:** Atopic dermatitis, Children, Balneotherapy, Comano Thermal spring water

## Abstract

**Background:**

Several studies have investigated the efficacy of balneotherapy in atopic dermatitis (AD), including a pediatric open randomized clinical trial conducted at the Comano thermal spring water center, which showed a significant reduction in AD severity and an improvement of the quality of life. However, so far many studies on balneotherapy in pediatric AD have included relatively small populations without identifying patients’ characteristics associated with their response. The aim of the present study was to identify any features associated with the clinical response to the Comano thermal spring water balneotherapy in a large cohort of pediatric AD patients.

**Methods:**

An observational study was conducted on 867 children aged ≤16 years (females 50.5%, mean patient’s age 5.9 years, standard deviation ±3.6 years) with mild to severe AD who underwent balneotherapy at the Comano thermal spring water center (Comano, Trentino, Italy) from April to October 2014. Patients were stratified according to their disease severity, which was evaluated using five SCORing Atopic Dermatitis (SCORAD) categories before and immediately after a thermal spring water balneotherapy course. Potential characteristics associated with the patients’ clinical response to Comano thermal spring water balneotherapy were investigated.

**Results:**

A statistically significant improvement in AD severity was observed after Comano thermal spring water balneotherapy (*p* < 0.0001). A significantly higher percentage of patients achieving improvement in AD severity was reported among children ≤4 years old (*p* < 0.0001) with early-onset AD (*p* < 0.0001), severe AD (*p* < 0.0001) or coexistent reported food allergies (*p* < 0.01). The therapy was well tolerated, and no relevant adverse effects were reported during the treatment course.

**Conclusions:**

Comano thermal spring water balneotherapy is a safe complementary treatment for pediatric patients with AD, as it was able to reduce the disease severity, especially in children ≤4 years old, with early onset AD, severe AD or concomitant food allergies.

## Introduction

With a prevalence of 15–20% in developed countries [1], atopic dermatitis (AD) is the most common chronic inflammatory skin disease in Europe and North America. Its lifetime prevalence in Italy among 9-year-old children was reported to be 15.2% [2].

AD typically arises first in pediatric age (within the first year of life in 60% of cases [3]). The skin eruptions consist of erythematous xerotic plaques with ill-defined borders which, in the acute phase, may be characterized by exudation, vesiculation and crusting. In the chronic phase, instead, skin thickening with lichenification is the predominant clinical sign. Bacterial superinfection of the lesions is common and - less frequently - viral and mycotic infections might occur. Since eczematous lesions are associated with intense pruritus and discomfort, AD has a considerable impact on the children’s quality of life, as it may lead to sleep disturbances, poor performance at school and psychological stress [4, 5].

As to its pathogenesis, AD is a skin immune-mediated disease with skin barrier alterations. The reduced barrier function is responsible for the penetration of environmental factors that, in turn, leads to local and systemic immune dysregulation [6]. Notably, AD is a highly heterogeneous disease with several clinical presentations and underlying molecular mechanisms. This clinical condition may vary in relation to the disease severity, chronicity, the patient’s age, their ethnic origin, IgE levels, filaggrin expression status, immune polarization (Th2/Th17/Th22) or microbiome alterations [7].

AD treatments include educational therapy [8], topical therapies such as emollients, topical corticosteroids (TCS) [9, 10], topical calcineurin inhibitors (TCI) [11] and crisaborole [12] as well as systemic therapies [13] like systemic steroids, cyclosporin and, more recently, dupilumab. Balneotherapy (i.e., body immersion in thermal water) could be a valuable option for pediatric patients with AD patients who seek “natural” complementary treatments, as acknowledged by a recent Italian consensus conference on the management of AD [14].

Several published works have studied balneotherapy in various skin diseases, including plaque psoriasis and AD, although many studies on balneotherapy in pediatric AD included relatively small populations. An open randomized clinical trial on 104 children with mild to moderate AD conducted at the Comano thermal spring water center in 2011 showed a significant reduction in AD severity and an improvement in life quality scores after Comano thermal spring water balneotherapy (*p* < 0.01 for both) [15]. In the latter study, patients who underwent Comano thermal spring water balneotherapy experienced a lower number and duration of AD relapses compared to the TCS-treated control group following the water treatment (*p* = 0.001), although the follow-up period was only 4 months.

With our study, we aimed to identify the subgroups of pediatric AD patients that could benefit the most from Comano spring water balneotherapy. The goal was to recognize the patients’ features associated with the clinical response to this treatment.

## Methods

This observational study was conducted at the Comano thermal spring water center (Comano, Trentino, Italy) from April to October 2014. Patients aged ≤16 years with previous doctor-confirmed diagnosis of AD based on the criteria of Hanifin and Rajka [16] were deemed eligible for inclusion in the study. The exclusion criteria encompassed comorbidities which could interfere with or contraindicate balneotherapy according to the principles of good clinical practice in thermal medicine and to the center’s policy [17, 18]: cutaneous diseases (e.g., fungal, viral or bacterial infections, skin cancer or ulcers) as well as any coexisting systemic pathologies (e.g., infections, heart diseases, immunodeficiencies or malignancies). An informed, written consent was obtained from all parents/guardians before including the patients in the study.

At admission, demographic and clinical data were collected through doctor interview and physical exam, including the onset age of AD, the history of AD in first-grade relatives, exposure to passive smoking, coexisting reported inhalant or food allergies. AD severity was recorded during the admission visit using the following five SCORAD (SCORing Atopic Dermatitis) categories: 0–15, 16–30, 31–40, 41–60, > 60. All the physicians who took part in the study had previously undergone formal training on how to use the SCORAD scale. The SCORAD score includes three items: percentage of the affected skin surface area (A; 0–100), intensity (B; 0–3 points are assigned to each of the following elements: erythema, edema/papulation, oozing/crusting, excoriation, lichenification, dryness) and subjective items (C; 0–10 points each of the following elements: pruritus and sleeplessness). Scores from individual items are then combined according to the following formula: A/5 + 7B/2 + C. The maximum and minimum SCORAD are 103 and 0, respectively. Mild AD is defined by SCORAD 0-15, moderate AD by SCORAD between 16 and 40 while SCORAD greater than 40 defines severe AD. After the completion of the balneotherapy course, at a 1- or 2-week follow-up visit, the same physician performed a second disease severity assessment through the same five AD severity categories. Based on the clinical findings, he or she prescribed the appropriate home treatment tailored on the clinical severity of their disease and provided patients with information on the available AD therapy options and the daily management of the disease. Pre- and post-Comano thermal spring water balneotherapy disease severity was compared both in the study population as a whole and in subgroups identified by the abovementioned demographical and clinical variables. The outcome after balneotherapy was defined “stable” if the disease severity before and after balneotherapy belonged to the same AD severity category, whereas it was defined “worsening” and “improving” if the disease severity shifted respectively to a higher or lower AD severity category.

Balneotherapy at the Comano thermal spring water center consists of 2–20 min of full body immersion in individual bathtubs. One to two baths are administered daily for a total course of 12–24 baths over a 1- or 2-week time-window, as this therapy can be individualized by administering shorter or less frequent baths to younger children and patients with more severe dermatitis. Pediatric patients were asked to refrain from any AD topical or systemic treatment except for emollients, that patients were instructed to apply once a day immediately after bathing. All patients were encouraged to take part in educational meetings held by a pediatrician or a dermatologist (named “School of Atopy project”) where AD physiopathology, diagnosis, management and treatments were discussed. The water used for balneotherapy at the Comano thermal spring water center springs at a temperature of 27.7 °C from the Comano Source, located 400 m above sea level, immediately adjacent to the thermal center. Among its chemical characteristics [19], it is worth mentioning a low overall saline content (dry residual 190 mg/l), with relatively high concentrations of bicarbonates (196.56 mg/l), calcium (48.90 mg/l) and magnesium (12.16 mg/l).

Statistical aggregated data processing was performed using SAS software (SAS institute, Cary, North Carolina, United States of America). Qualitative data were presented through histograms and/or as frequency tables (number of observations or percentages). Quantitative data were expressed as number of observations, mean and standard deviation (SD). The associations between AD severity, expressed through SCORAD categories, and qualitative variables were evaluated using the chi-squared test with Yates correction. A statistically significant difference was defined by a *p*-value ≤0.05. Pre- and post-balneotherapy AD severity were compared using the Wilcoxon signed rank sum test (significant if *p*-value ≤0.05). To analyze whether some variables were associated with better outcomes after Comano spring water balneotherapy, multivariate logistic regression analysis of the probability of improvement was performed, expressed as odds ratios (OR) and their relative 95% confidence intervals (95% CI). 

## Results

867 Italian patients affected by AD were included in the study (Table 1). Their mean age was 5.9 years with a SD of ±3.6 years and 50.5% of them were females. Before Comano thermal spring water balneotherapy, 41.2% were affected by mild AD (SCORAD 0–15), 43.6% by moderate AD (SCORAD 16–40) and 15.2% by severe AD (SCORAD > 40).

**Table 1:** Baseline characteristics of the patients included in the study population

Characteristics (*n* = 867)	
Females	438 (50.5%)
Age (years) ± SD	5.9 ± 3.6
Age of onset of AD	
< 2 months	229 (26.4%)
2–6 months	242 (27.9%)
7–12 months	154 (17.8%)
13-36 months	149 (17.2%)
> 36 months	93 (10.7%)
History of AD in a first-grade relative	35 (4.0%)
Exposure to passive smoking	134 (15.5%)
Reported inhalant allergy	199 (23.0%)
Reported food allergy	82 (9.5%)
SCORAD	
0–15	357 (41.2%)
16–40	378 (43.6%)
> 40	132 (15.2%)

*AD* atopic dermatitis, *SCORAD* SCOring Atopic Dermatitis, *SD* standard variation

Balneotherapy was well tolerated. No relevant adverse effects were reported during the treatment course. Post-balneotherapy AD severity significantly improved compared to pre-balneotherapy severity (*p* < 0.0001).

**Fig. 1:**
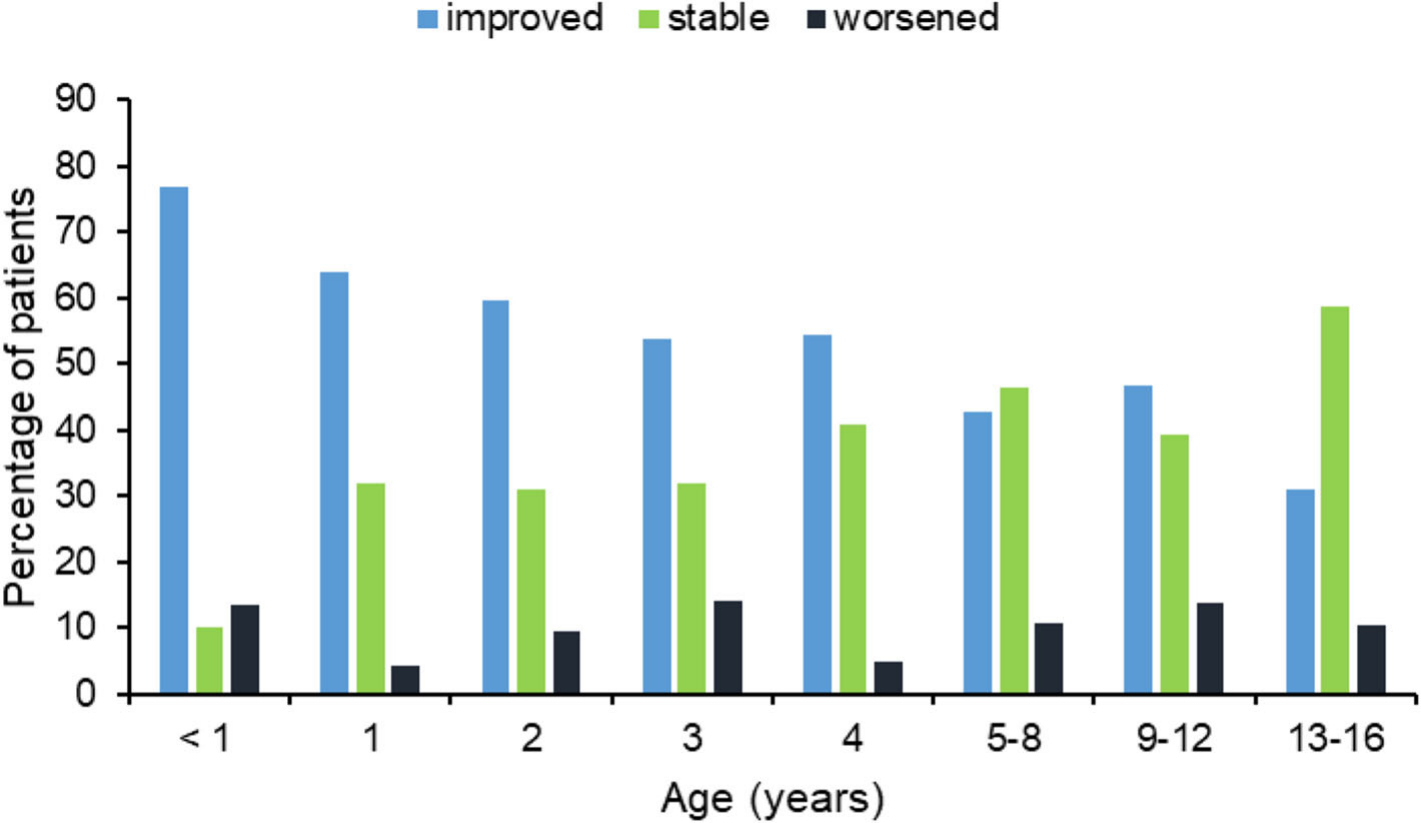
Response to balneotherapy according to patient age. Differences between pre- and post-balneotherapy disease severity were expressed as improved, stable or worsened (*p* < 0.0001)

An association between the patients’ age and the AD severity improvement was observed (*p* < 0.0001 when comparing children ≤4 years old vs > 4 years old; Fig. 1). While 76.7% of children younger than 1 year of age experienced a reduction in AD severity, the percentage in older children was significantly lower. An inverse trend between percentage of patients improving after balneotherapy and age was noticed (63.7% of 1-year-old children, 59.5% of 2-year-olds, 53.9% of 3-year-olds, 54.3% of 4-year-olds, 43.7% of children older than 4 years and 31.0% of 13–16-year-old-children). On the contrary, the stable disease severity rate after balneotherapy was higher among older children (10.0% of children younger than 1 year, 31.9% of 1-year-olds, 31.0% of 2-year-olds, 32.1% of 3-year-olds, 40.7% of 4-year-olds, 44.3% of children older than 4 years and 58.6% of children aged 13–16 years). Moreover, the percentage of children whose AD worsened was not related to their age: 13.3% of children younger than 1 year, 4.4, 9.5, 14.1, 4.9 and 11.9% of children aged respectively 1, 2, 3, 4 years and older.

**Fig. 2:**
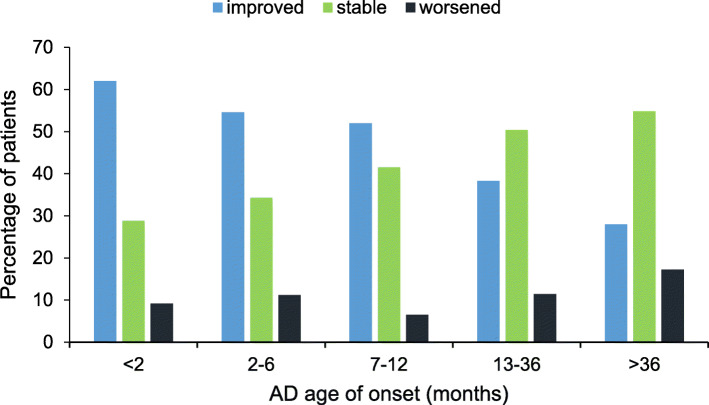
Response to balneotherapy according to AD age of onset. Differences between pre- and post-balneotherapy disease severity were expressed as improved, stable or worsened (*p* < 0.0001)

**Fig. 3:**
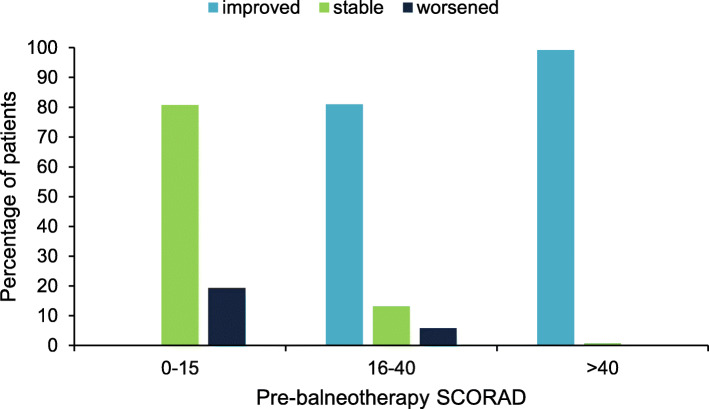
Response to balneotherapy related to basal AD severity at admission. Differences between pre- and post-balneotherapy disease severity were expressed as improved, stable or worsened (*p* < 0.0001) *SCORAD *SCORing Atopic Dermatitis

The onset age and basal severity of AD were also significantly associated with AD severity improvement (*p* < 0.0001 and *p* < 0.0001, respectively). The percentage of patients responding to balneotherapy was, indeed, significantly lower among children with later onset of AD: an improvement was observed in 56.6% of children who developed AD within the first year of life, as opposed to 28.0% of children whose AD arose after the age of 3 years (Fig. 2). Furthermore, as far as AD severity at admission is concerned, greater disease severity was associated with response in a higher proportion of patients (Fig. 3). For example, AD severity improved in 99.2% of patients with severe AD, as opposed to 41.6% in those with mild to moderate disease (*p* < 0.0001 for SCORAD ≤40 vs > 40). Stable disease severity in patients with severe, moderate and mild AD was noticed in 0.8, 13.2 and 80.7%, respectively. Finally, a deterioration of AD severity was reported in 19.3% of patients with mild disease and 5.8% of those with moderate disease.

**Fig. 4:**
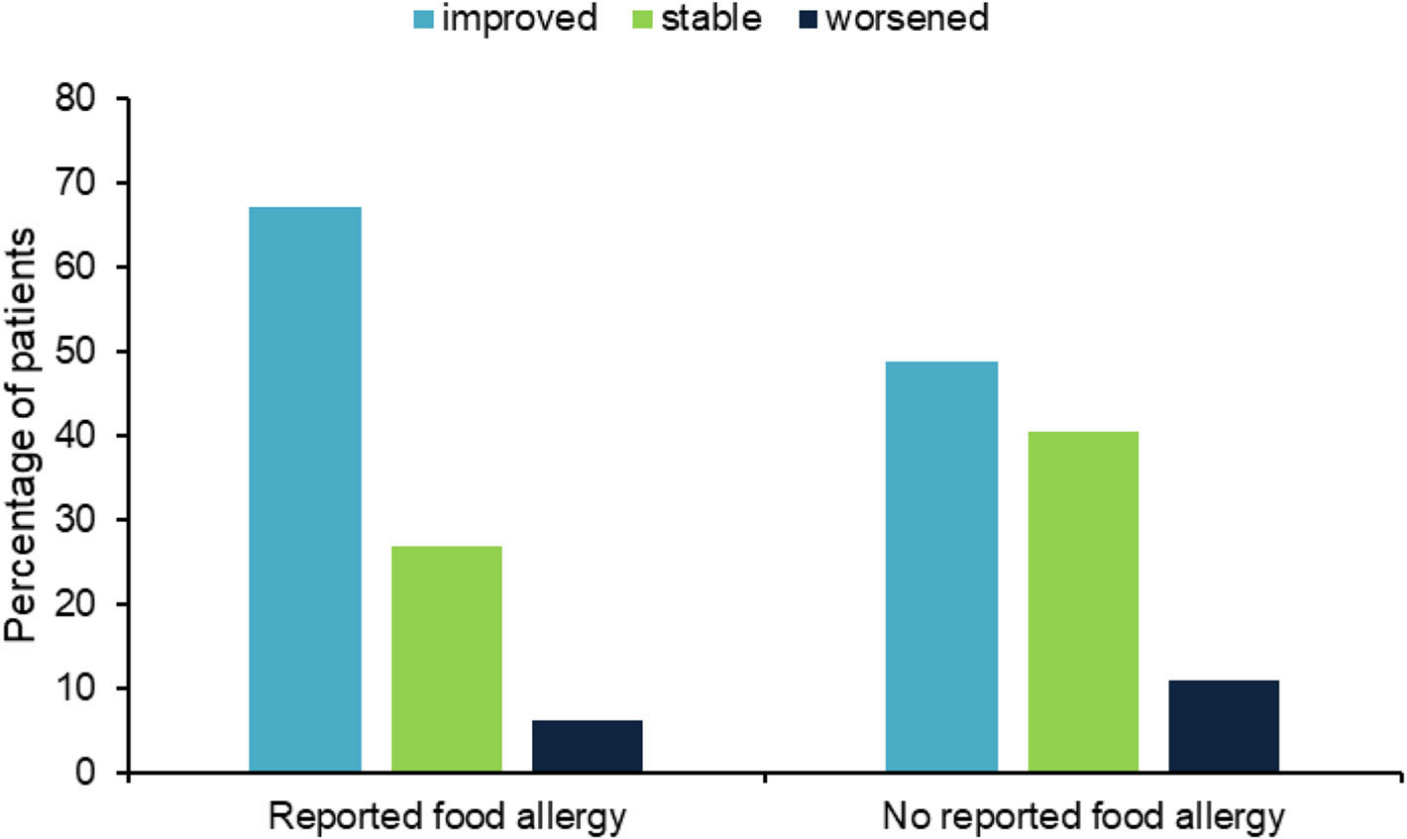
Response to balneotherapy related to patient reported food allergy. Differences between pre- and post-balneotherapy disease severity were expressed as improved, stable or worsened (*p* < 0.01)

Reported food allergies also showed an association with the percentage of patients experiencing improvements of AD severity (*p* < 0.01; Fig. 4), while no statistically significant association emerged with passive smoking (*p* = 0.82) and reported inhalant allergy (*p* = 0.51).

These results were further tested through a multivariate logic regression analysis on the probability of AD severity improvement after balneotherapy (Table 2). Such multivariate analysis showed that younger children are far more likely to achieve an improvement in AD severity than older children. While this observation was supported by statistically significant OR values in all age groups (OR 1.72; CI 1.26–2.35 for children ≤4 years old vs > 4 years old), greater evidence was found for children 1-year-old or younger (OR 3.11; CI 1.26–7.69 for children aged less than 1 year compared to children aged more than 4 years). Significant OR values were also noticed in patients with early-onset AD and concomitant presence of reported food allergy (OR 0.79; CI 0.71–0.89 and OR 1.68; CI 1.02–2.78, respectively). On the contrary, multivariate analysis did not show any significant OR values in patients with passive smoking and reported inhalant allergy.

**Table 2:** Results of the multivariate logistic regression analysis on the probability of disease severity improvement after balneotherapy

Variable	OR	95% CI	*P*-value
Age (< 1-year-old vs > 4-year-old)	3.11	1.26 7.69	0.013
Age (≤ 4-year-old vs > 4-year-old)	1.72	1.26 2.35	0.0007
Age of onset of AD	0.79	0.71 0.89	< 0.0001
History of AD in a first grade relative	1.79	0.83 3.90	0.12
Passive smoking	1.08	0.74 1.59	0.71
Reported inhalant allergy	1.26	0.85 1.87	0.28
Reported food allergy	1.68	1.02 2.78	0.003

*AD* atopic dermatitis

## Discussion

A considerable number of adult and pediatric patients across several countries access balneotherapy centers for treating skin diseases – mainly psoriasis and eczema. The efficacy of the Comano thermal spring water center was studied in plaque psoriasis [20–22] as well as AD [15]. As mentioned above, Comano thermal spring water balneotherapy was studied specifically for the latter disease in a recent open randomized clinical trial, where it demonstrated its efficacy and safety [15].

In our study, we aimed to identify the subgroups of pediatric AD patients that could benefit the most from Comano thermal spring water balneotherapy. The goal was to recognize the patients’ features associated with the clinical response to this treatment. Such information, which had not been investigated in any previous studies on balneotherapy, may bring clinical relevance to the physicians dealing with AD patients who seek complementary treatments. A statistically significant reduction in AD severity after balneotherapy was observed. Moreover, a statistically significant higher percentage of patients – among children ≤4 years old, with early-onset AD, severe AD or concomitant reported food allergy – achieved an improvement in AD severity, while no association emerged with passive smoking and reported inhalant allergy.

It is worth mentioning that the use of balneotherapy for treating AD has been the subject of a number of clinical studies in various other centers. In a study on AD patients conducted at the Dead Sea [23, 24], clearance of skin lesions was observed on 89 and 97% of them following 4 and 6 weeks of balneotherapy, respectively. Moreover, another study [25] has showed that bathing in a Death Sea salt solution can improve skin barrier function and skin hydration as well as reduce inflammation in adult atopic dry skin. Balneotherapy was also reported to be effective for AD in studies conducted at La Roche-Posay Thermal Center [26] and Avène hydrotherapy center [27].

Several factors may be involved in explaining the efficacy of the Comano thermal spring water balneotherapy. A first hypothesis is that thermal water may exert an anti-inflammatory action by interfering with cytokine production and secretion in keratinocytes. Such process was extensively analyzed through in vitro studies on cultured psoriatic keratinocytes. In those cells, thermal water was shown to interfere with IL-6 production and secretion, cytokeratin 16 expression [28], Tumor Necrosis Factor (TNF)-alpha expression, IL-8 production and secretion [29] and with Vascular Endothelial Growth Factor-A expression and secretion [30]. Although these studies were conducted on psoriatic keratinocytes only, it appears plausible that a similar immunomodulatory action may also occur on AD-affected skin. Interestingly, inflammatory biomarkers correlated with AD severity – such as high-morbility group box 1 (HMGB1) – have been recently identified [31] and they may be useful in future studies to evaluate the response to balneotherapy. Regarding a second hypothesis, genomic and metagenomic studies were conducted on the Comano thermal spring water in order to ascertain its microbiome composition [32]. It emerged that Comano thermal water’s microbiome includes the *Sphingomonadales, Rhizobiales*, and *Caulobacterales* orders as well as the *Bradyrhizobiaceae* and *Moraxellaceae* families. A comparative genomic analysis of 72 isolates and 30 metagenome-assembled genomes (MAGs) showed that most isolates and MAGs belonged to new species or higher taxonomic ranks widely distributed in the microbial tree of life. At the same time, recent evidence from the relevant literature seems to indicate a potential role played by the skin microbiome in the pathogenesis of AD. About 90% of AD patients show lesional skin colonization by *Staphylococcus aureus (S. aureus)*, a bacterium which would not be found on the skin of the majority of healthy subjects [33]. Interestingly, *S. aureus* colonization is associated with AD severity and flares. Furthermore, a relative absence of commensal bacterial strains producing antimicrobial activity against *S. aureus* was observed in AD patients [34]. It is clear that further studies are required in order to gain better understanding of the skin microbiome in the pathogenesis of inflammatory skin diseases. Nonetheless, a potential role of the thermal water’s microbiome in explaining the Comano thermal spring water effect remains an intriguing hypothesis that needs to be clinically validated through an assessment of pre- and post-balneotherapy skin microbiota associated to the clinical outcome of the treatment. Interestingly, a recently isolated microorganism from Avène thermal spring water, *Aquaphilus dolomiae*, was shown to have regulatory activity on the inflammatory and immune responses in AD keratinocyte models [35]. Finally, in order to explain our findings, there may be an influence on AD due to the patients’ exposure to a different environment with different temperature, humidity, presence of allergens and air pollution. The absence of psychological stressors may play a role as well.

A potential hypothesis that may help shed light on why some subgroups of AD patients in our study achieved a different response to balneotherapy is the fact that they may have different underlying pathogenic mechanisms. For example, it has long been known that non-allergic and IgE-associated forms of AD develop differently in terms of cell and cytokine patterns in peripheral blood and affected skin [36].

Also, various AD endotypes were described based on differences in patient age and race, AD severity and course (chronic vs acute), immune polarization of T cell subsets, interleukin and chemokine expression, IgE levels, skin barrier defects (e.g., altered lipid composition, expression status of filaggrin, keratin 16, locrin, periplakin) and microbiome alterations [7]. The definition of such AD endotypes has been a crucial advance in the understanding of AD, paving the way to a tailored therapeutic approach, and it may also play a role in explaining the different outcomes after balneotherapy of different subsets of patients. For example, while Th2 axis activation seems to represent a common denominator in AD, an increased Th17 axis is characteristically observed in pediatric, elderly, intrinsic and Asian AD [7]. Moreover, some immunological differences also seem to be related to AD severity [37], the patients’ age [38], skin barrier defects (caused by both inheritable and environmental factors) and skin microbiome alterations. Skin barrier impairment is a crucial feature of AD. In the scientific literature, there is growing evidence that impaired skin barrier and low-dose cutaneous allergen exposure can even lead to allergen sensitization through the skin and food allergy, according to the “dual-allergen exposure hypothesis” [39–43]. According to our post-balneotherapy data, the disease severity is more likely to improve in patients with reported food allergy. However, future studies are needed to target this specific population in order to elucidate a potential mechanism which could explain the latter phenomenon.

Our study was limited by the absence of a control group and follow-up data, which is mostly connected to the clinical setting in which it was carried out, i.e., visits for balneotherapy. Furthermore, collecting follow-up data would not have been feasible, since the vast majority of the enrolled patients live far away from the Comano thermal spring water center. Finally, the different treatments patients underwent before Comano thermal spring water exposure may have been a confounder. However, the large study population size, the availability of pre- and post-treatment evaluations and multivariate analysis are the strong points of this study, which succeeded in pointing out with statistical significance an improvement in AD severity, without relevant adverse effects during the treatment course, as well as the characteristics associated with the clinical response to Comano thermal spring water balneotherapy.

## Conclusions

Comano thermal spring water balneotherapy is a safe complementary treatment for pediatric patients with AD, which demonstrated a reduction in terms of severity especially among children ≤4 years old, with early-onset AD, severe AD or concomitant food allergies.

## Data Availability

The access to datasets generated and/or analyzed during the current study is not publicly available due to the Center’s research policy to guarantee the privacy of the participants, whose research data are confidential. Aggregate analyses are however available on reasonable request to the corresponding author.

## Abbreviations

AD: Atopic Dermatitis
CI: Confidence Interval
HMGB1: High-Morbidity Group Box 1
IL: Interleukin
MAG: Metagenome-Assembled Genomes
OR: Odds Ratio
SCORAD: SCOring Atopic Dermatitis
SD: Standard Deviation
TCI: Topical Calcineurin Inhibitors
TCS: Topical Corticosteroids
TNF: Tumor Necrosis Factor
VEGF-A: Vascular Endothelial Growth Factor A
